# Simultaneous activation of multiple vestibular pathways upon electrical stimulation of semicircular canal afferents

**DOI:** 10.1007/s00415-020-10120-1

**Published:** 2020-08-10

**Authors:** Anissa Boutabla, Samuel Cavuscens, Maurizio Ranieri, Céline Crétallaz, Herman Kingma, Raymond van de Berg, Nils Guinand, Angélica Pérez Fornos

**Affiliations:** 1 Division of Otorhinolaryngology Head and Neck Surgery, Geneva University Hospitals and University of Geneva, Geneva, Switzerland; 2 Division of Balance Disorders, Department of ENT, Maastricht University Medical Centre, Maastricht, The Netherlands; 3 Faculty of Physics, Tomsk State Research University, Tomsk, Russia

**Keywords:** Bilateral vestibulopathy, Vestibular implant, Vestibulo-ocular reflex, Vestibulo-spinal reflex, Neuroprosthesis, Electrical stimulation

## Abstract

**Background and purpose:**

Vestibular implants seem to be a promising treatment for patients suffering from severe bilateral vestibulopathy. To optimize outcomes, we need to investigate how, and to which extent, the different vestibular pathways are activated. Here we characterized the simultaneous responses to electrical stimuli of three different vestibular pathways.

**Methods:**

Three vestibular implant recipients were included. First, activation thresholds and amplitude growth functions of electrically evoked vestibulo-ocular reflexes (eVOR), cervical myogenic potentials (ecVEMPs) and vestibular percepts (vestibulo-thalamo-cortical, VTC) were recorded upon stimulation with single, biphasic current pulses (200 µs/phase) delivered through five different vestibular electrodes. Latencies of eVOR and ecVEMPs were also characterized. Then we compared the amplitude growth functions of the three pathways using different stimulation profiles (1-pulse, 200 µs/phase; 1-pulse, 50 µs/phase; 4-pulses, 50 µs/phase, 1600 pulses-per-second) in one patient (two electrodes).

**Results:**

The median latencies of the eVOR and ecVEMPs were 8 ms (8–9 ms) and 10.2 ms (9.6–11.8 ms), respectively. While the amplitude of eVOR and ecVEMP responses increased with increasing stimulation current, the VTC pathway showed a different, step-like behavior. In this study, the 200 µs/phase paradigm appeared to give the best balance to enhance responses at lower stimulation currents.

**Conclusions:**

This study is a first attempt to evaluate the simultaneous activation of different vestibular pathways. However, this issue deserves further and more detailed investigation to determine the actual possibility of selective stimulation of a given pathway, as well as the functional impact of the contribution of each pathway to the overall rehabilitation process.

## Introduction

Bilateral vestibulopathy, a bilaterally reduced or absent vestibular function, causes disabling symptoms such as imbalance and oscillopsia. It has a direct impact on the quality of life in at least three million people worldwide [1, 2]. Currently, this pathology remains poorly understood and treatment options are limited [3, 4]. Our group attempts to develop a treatment alternative for these patients: a vestibular implant. The concept is to use electrical currents to transmit head motion information normally detected by the vestibular system, to the vestibular nerve [5, 6]. This is comparable to the concept of the cochlear implant for hearing rehabilitation. Briefly, the vestibular implant comprises an implanted neural stimulator incorporating electrodes to be positioned close to the branches of the vestibular nerve [5]. The stimulator applies electrical currents through these vestibular electrodes that can be modulated in intensity or rate using motion sensors attached to the patient’s head. The electrical activity delivered by the device thus signals the speed and direction of head movements, replacing the damaged vestibular system [7].

The feasibility of this concept was first established in several animal studies, starting in the 1960s [8, 9]. More recent feasibility studies using animal models demonstrated the effectiveness of using chronically implantable devices to generate controlled vestibulo-ocular responses [10–13]. Special stimulation paradigms were developed to improve outcomes (e.g., peak eye velocities, alignment) [10–13]. Other studies investigated fundamental aspects of vestibular function such as adaptation and plasticity [14, 15], as well as how the different vestibular reflexes complement each other to achieve complex behavior (e.g., contribution of the vestibulo-ocular and vestibulo-spinal reflexes to gaze stabilization) [16]. Since then, other approaches have been developed to expand the original approach targeting semicircular canals exclusively to also attempt stimulation of the otolith organs [17]. Human research also made important progress in recent years [18, 19]. Special implantable stimulators were developed and were progressively improved, together with the surgical approach for optimal and safe implantation of the device [20–23]. The most recent results on human patients demonstrated that it is possible to restore vestibular reflexes [24–28], to significantly improve visual abilities in dynamic conditions (i.e., while walking) [29] and to generate controlled postural responses in implanted patients [30, 31]. Other studies demonstrated the feasibility of using long-term electrical stimulation to stimulate the vestibular pathways, both with systems designed for stimulation of the semicircular canals or the otolithic organs [17, 27, 32]. Taken together, these results pave the way for the clinical application of the vestibular implant.

The optimization of outcomes achieved with the vestibular implant requires that the multidimensional nature of the “balance system” is considered. After all, balance is an important “sixth sense” which substantially relies on the parallel activation of multiple vestibular pathways. These pathways start at the peripheral vestibular system and project to the vestibular nuclei in the brainstem. These nuclei are the neural relay of the vestibular pathways where information from other sensory systems (i.e., vision, proprioception) also converge. The central neurons of the vestibular nuclei project to different structures that generate reflexes for gaze stabilization (vestibulo-ocular reflex) [33] and for postural control during everyday activities (vestibulo-collic and vestibulo-spinal reflexes) [34–36]. Finally, vestibulo-thalamo-cortical (VTC) pathways carry information to the brain which combines vestibular and extra-vestibular cues to ensure more complex central functions, such as self-motion perception or spatial navigation. While previous studies have addressed each of these pathways individually, the purpose of this study was to simultaneously explore the relationships between the electrical stimulus delivered to the vestibular system and the resulting responses of the different vestibular pathways. Specifically, we attempted to investigate the main characteristics and the amplitude growth function (amplitude of the response as a function of stimulation current) of three vestibular pathways: vestibulo-ocular reflex (VOR), vestibulo-collic reflex (VCR), and vestibulo-thalamo-cortical (VTC) elicited with a set of controlled electrical stimuli. This study follows up on our previous investigations attempting to determine the most efficient stimulation paradigm to optimize outcomes with the vestibular implant [37]. Shorter phase durations (< 200 µs) and, to a lesser extent, slower pulse rates (< 200 pulses-per-second, pps) allowed maximizing the electrical dynamic range available for eliciting a wider range of intensities of vestibular percepts. Interestingly, however, this observation was not consistent for VOR responses. In this case, the main factor allowing to maximize the response was modulation depth (i.e., amplitude of the modulation signal), while variations in phase duration and pulse rate appeared less effective than previously reported in animals [11, 13]. The present study attempts to go one step further, and assess the variability of responses of different pathways elicited simultaneously using different stimulation profiles.

## Methods

### Subjects, device and surgery

Three bilateral vestibulopathy patients who previously received a vestibular implant prototype [7], participated in this study. Details on the inclusion criteria, device and surgical procedures can be found in previous publications [5, 7, 24]. Briefly, the device consisted of a modified cochlear implant (MED-EL, Innsbruck, Austria) providing one to three extra-cochlear electrodes for vestibular stimulation (Table 1). These vestibular electrodes were implanted in the vicinity of the lateral, posterior and superior ampullary branches of the vestibular nerve (respectively, LAN, PAN and SAN) using an intralabyrinthine or extralabyrinthine surgical approach [20, 23, 38]. Note that the PAN electrodes in patients S1 and S2 were not tested during the experiments presented here (grayed out in Table 1). Stimulation with these electrodes did not evoke any vestibular responses even at the highest current levels available for safe stimulation. This is probably due to the traumatic etiology of these cases (temporal bone fracture going through the ampulla of the PAN).

**Table 1:** Main demographic characteristics of the three patients participating in this study

Patients	Sex	Etiology	Onset	Age at implantation	Year implanted	Implanted side	Vestibular electrodes	Surgical approach
S1	F	Traumatic	Acute (< 1 year)	67	2013	Left	PAN/LAN/SAN	IL
S2	M	Traumatic	Acute (3 years)	53	2015	Right	PAN/LAN/SAN	IL
S3	M	Congenital/idiopathic	Progressive	46	2008	Left	PAN	EL

*M* male, *F* female, *PAN* posterior ampullary nerve, *LAN* lateral ampullary nerve, *SAN* superior ampullary nerve, *EL* extralabyrinthic [20], *IL* intralabyrinthic [21]

All patients were recruited at the Division of Otorhinolaryngology and Head and Neck Surgery of the Geneva University Hospitals. Note that only one vestibular electrode was activated at a time for a given experimental trial. All cochlear electrodes were switched off during the experimental procedures.

### Electrical stimulation

The setup for the electrical stimulation was composed of a computer running custom software based on MATLAB R2014b (The Mathworks Inc., Natick, Massachusetts, USA). This software allowed customization of stimulation parameters (current intensity, pulse rate, phase width, electrode, current range, train pulse characterization, etc.). The computer communicated this information to the implanted stimulator via a special interface device (dRIB; MED-EL, Innsbruck, Austria) and the system’s antenna.

Each experimental trial consisted of 100 electrical stimuli presented at a repetition rate of 5 Hz. The electrical stimuli involved one or several cathodic-first, biphasic, charge balanced pulses delivered to the vestibular nerve with one of the implanted vestibular electrodes. First, we investigated the growth function of the responses of the three vestibular pathways (VOR, VCR, and VTC), using a single pulse with 200 µs phase duration (S1-SAN, S1-LAN, S2-SAN, S2-LAN and S3-PAN). Second, we compared the growth functions obtained with three different stimulation paradigms on one subject (S1-SAN and S1-LAN) who was available for this additional experiment. The three stimulation paradigms were (1) a single pulse with a phase of 200 µs (as used in the previous experiment and in our preceding studies [37]); (2) a single pulse with a short phase of 50 µs (similar to that commonly used in clinical cochlear implant fittings); and (3) a pulse train of four 50 µs/phase pulses presented at rate of 1600 pulses-per-second (pps) (total charge per stimulation trial equal to the single pulse with a 200 µs phase duration).

### Characteristics and growth functions of the VOR, VSR, and VTC pathways

First, a measurement without any electrical stimulation (0 µA) was performed to record baseline response levels (e.g., noise). Then consecutive experimental trials were performed with increasing current amplitude (steps of 50 µA) to investigate the characteristics and the growth functions of each vestibular response, up to the upper comfortable level (UCL). The UCL is defined to be the current level immediately below the level where undesired effects are observed (i.e., facial nerve activation, uncomfortably loud sound) or at the maximum safe current level allowed by the device, similar to our previous studies [7]. Note that this experimental design involving very short stimulation trials did not comprise special psychophysical paradigms to compensate for adaptation effects (e.g., ascending/descending) which would have resulted in increased experimental times. In our experimental conditions, the bias induced by increased experimental time would have been greater than that induced by any potential adaptation effects.

**Fig. 1:**
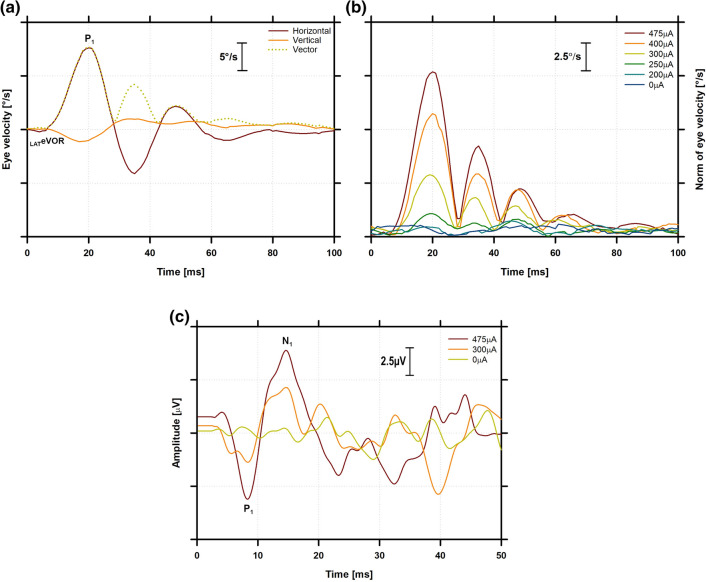
Example of recordings and data processing of results obtained in patient S1 (SAN electrode) upon stimulation with a charge-balanced, cathodic-first, biphasic current pulse of 200 µs per phase. **a** Horizontal, vertical, and vector norm components of the averaged eVOR signal (respectively, solid dark red, solid orange, and dotted green lines). _LAT_eVOR marks the beginning of the eye movement (i.e., the latency), and *P*_1_ marks the first peak of the total peak eye velocity vector (*P*_1_VOR). **b** Example of the evolution of the norm of the eye velocity vector (PEV) while applying increasing stimulation currents from 0 to 475 µA. **c** Average ecVEMP response. *P*_1_ and *N*_1_ mark the location of the first positive and second negative peak of the response, respectively, to stimulation currents ranging from 0 to 475 µA. Note that each of the panels represents a different response. Consequently, the vertical axes of each graph have different scales (see the scale bars in each panel)

Figure 1 shows an example of one of the recordings for S1 obtained upon electrical stimulation of the vestibular electrode implanted in the proximity of the SAN. The markers in the figure illustrate the different time points that were considered in the analysis of latency and amplitude of the different responses, explained in detail below.

Electrically evoked VOR responses (eVOR) were recorded using a binocular, video-based eye tracking system at a high sampling rate (1000 Hz) to allow acquisition of short-latency eye-movements (EyeLink 1000 Plus; SR Research, Ottawa, Canada). Some patients suffered from strabismus which hindered accurate binocular fixation; therefore, only the dominant eye was recorded in all subjects. Each experimental trial started with a calibration procedure which consisted of nine sequential fixations of a dot moving randomly around the computer screen borders, followed by a similar nine-point validation procedure to ensure calibration accuracy (error < 0.1°). Horizontal and vertical eye velocity data from the EyeLink system were imported to MATLAB R2018b. Peak eye velocity (PEV) of the signal was calculated as the square root of the sums of the squares of horizontal and vertical eye movements. Trials including artefacts (saccades or blinks) were manually removed. Then the average of artefact-free PEV responses was calculated. The latency of the electrically evoked vestibulo-ocular reflex (eVOR) is determined by the beginning of the eye movement, calculated as the first inflection point of the total PEV signal (_LAT_eVOR). The consecutive eVOR peaks were determined using the maximum or minimum, depending on signal polarity, of the second derivate of the total PEV signal (see Fig. 1a).

Electrical cervical vestibular evoked myogenic potentials (ecVEMPs) were recorded with the NeuroAudio system (Neurosoft, Ivanovo, Russian Federation), with the active recording electrodes positioned on the main belly of the sternocleidomastoid muscle (SCM), approximately equidistant from the mastoid process and the sternum. The ground electrode was placed on the superior part of the sternum, and the indifferent electrode on the forehead. Instead of being in the standard supine position and lifting the head, we had patients sit down with the head placed on a head support tower. The patient was requested to look straight ahead to a 24″ computer screen (XL2420-B; BenQ, Taipei, Taiwan) projecting a 12 mm-wide cross (eye-to-screen distance 63 cm). Sufficient SCM tension was obtained by having the patient turn the shoulders slightly. This non-standard patient configuration was necessary to allow simultaneous recording of eye movements and to limit patient fatigue resulting from repeated testing. ecVEMP results for each experimental trial were amplified, averaged, and imported into MATLAB R2018b (The Mathworks Inc., Natick, Massachusetts, USA). The signals were then low-pass filtered at 500 Hz using a ninth-order IIR filter with zero frequency shift. The latencies and amplitudes of the positive (*P*_1_) and negative (*N*_1_) peaks were determined using the Matlab function “findpeaks”. When multiple peaks were identified by this function for a given wave, the optimum peak was selected in consensus by four experienced clinical observers (authors AB, NG, SC, MR and APF). The latency of the ecVEMPs was calculated as the latency of the first peak of muscular contraction (*P*_1_), which corresponds to the initiation of the neck movement.

After each stimulation trial, the patient had to report the self-perceived intensity of the stimulus using the clinical 0–8 visual-analog scale (0—no perception, 8—too strong) used for fitting cochlear implant patients in our center. Patients were also asked to describe the percept. Only percepts that could be identified as vestibular in consensus between experimenters were considered (see also [7]). For example, percepts evoking motion or disorientation were included, while percepts evoking sound, pain, or tickling were not considered.

### Data analysis and statistics

All analyses were carried out with SigmaPlot 14 (Systat Software, San Jose, CA, USA) and will be presented in “Results”.

## Results

**Fig. 2:**
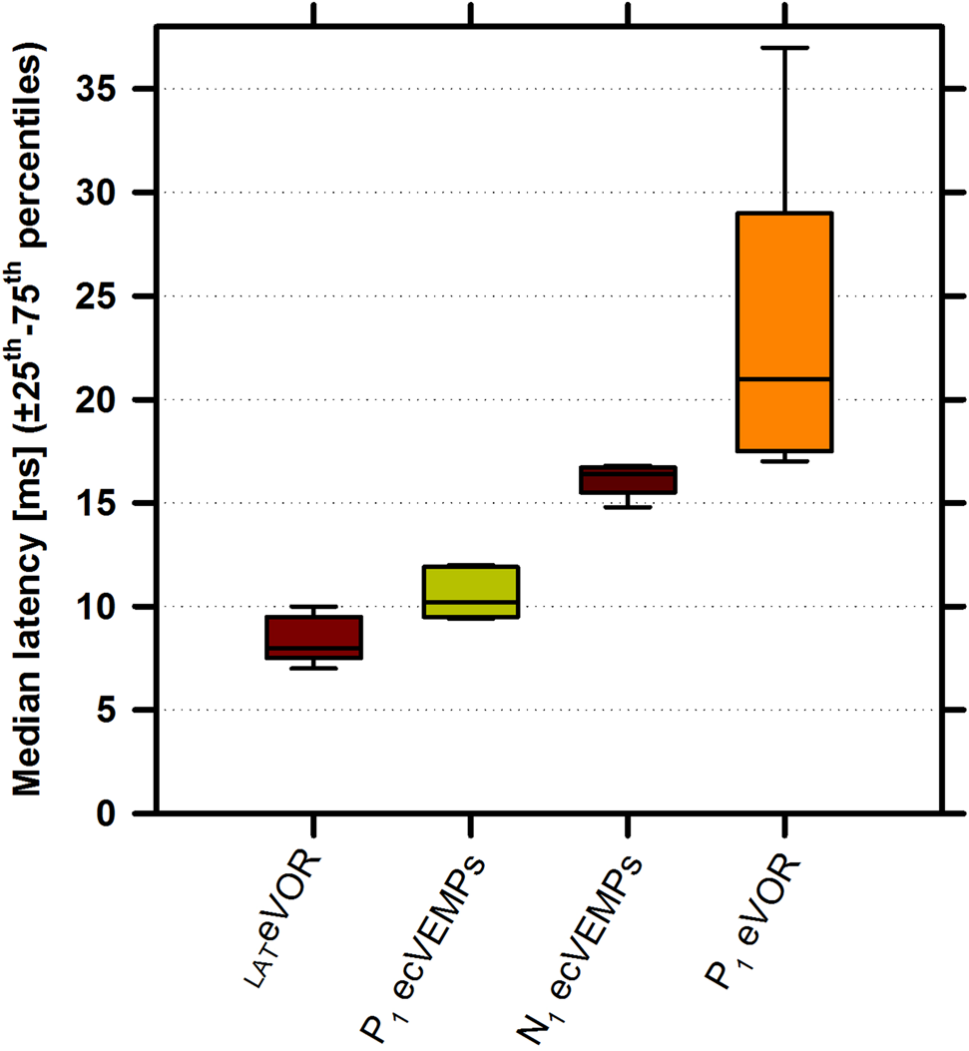
Latencies of the eVOR and ecVEMP responses elicited upon stimulation with a biphasic, charge-balanced, cathodic-first current pulse of 200 µs per phase at the UCL (see also Fig. 3). Box plots indicate median values, 25th and 75th percentile values (coloured boxes) as well as 10th and 90th percentile values (error bars) for all subjects and all electrodes tested. Three patients participated in this experiment (S1, S2 and S3), in whom a total of five electrodes were tested (S1-SAN, S1-LAN, S2-SAN, S2-LAN and S3-PAN)

**Fig. 3:**
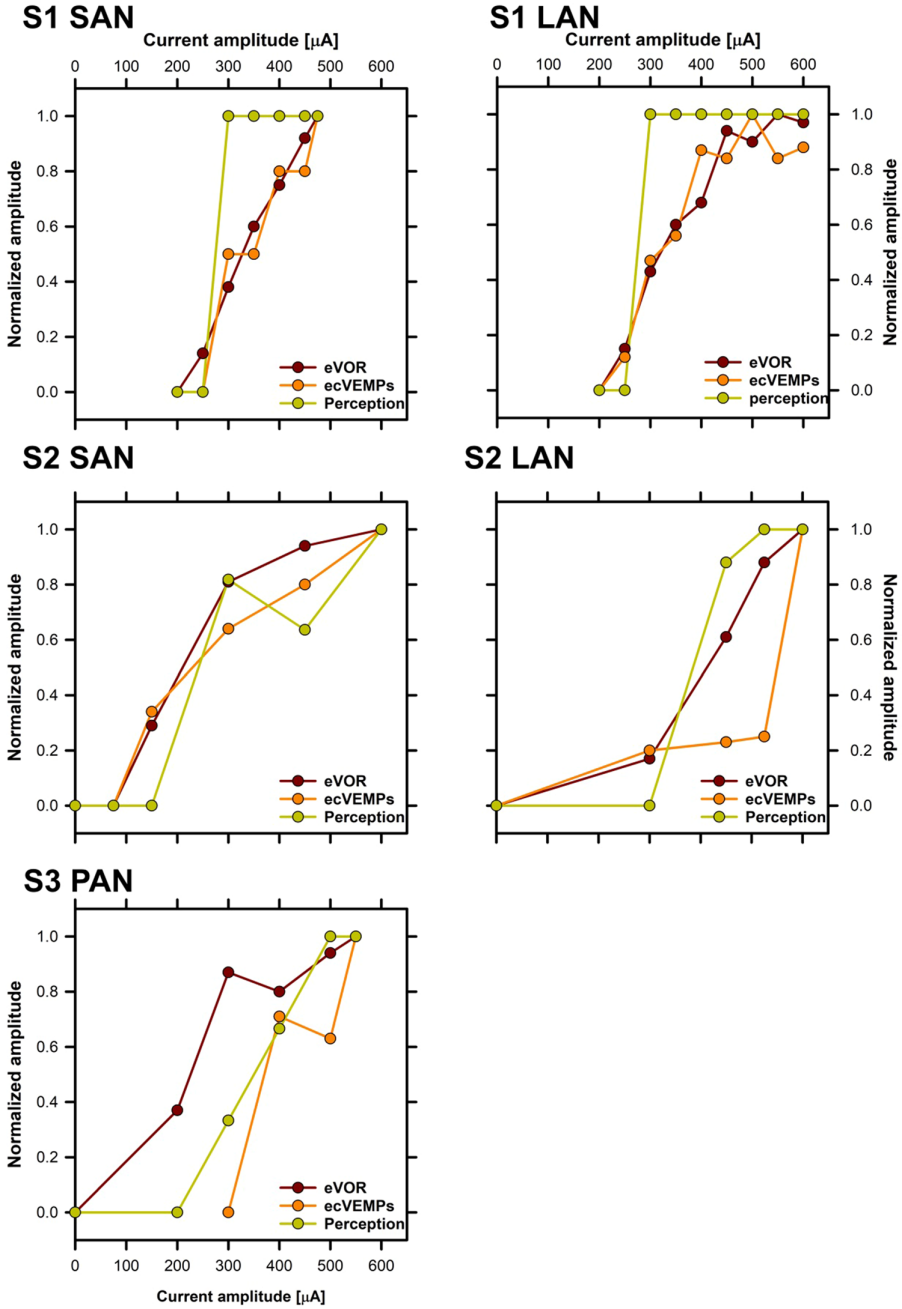
Growth function of the normalized amplitude of the P_1_ eVOR (red plot), the N–P amplitude for ecVEMPs (orange plot), and individual self-reported percept intensity (green plot) versus current amplitude. Each panel represents responses measured simultaneously in one subject and upon stimulation with one vestibular electrode, for a single-pulse stimulation paradigm, 200 µs phase width

Figure 2 compares the median latencies of the average eVOR and ecVEMP responses for trials with a single stimulating pulse of 200 µs phase width and maximum current intensity (see also Fig. 3 for individual growth functions). Five electrodes were tested in three participating patients. The fastest response observed was that of the _LAT_eVOR (median latency 8.0 ms). The ecVEMPs had a median latency of 10.2 ms. The *N*_1_ peak of the ecVEMPs appeared at median latency of 16.4 ms, and the *P*_1_ of the eVOR response at a median latency of 21.0 ms. Individual results and group medians (25th–75th percentiles) are presented in detail in Table 2.

**Table 2:** Main characteristics of eVOR and cVEMPs elicited upon stimulation with a charge-balanced, cathodic-first, biphasic current pulse of 200 µs per phase at the maximum intensity (see also Figs. 2 and 3)

eVOR					ecVEMPs		
Subject	Electrode	_LAT_eVOR [ms]	*P*_1_ latency [ms]	*P*_1_ amplitude [°/s]	*P*_1_ latency [ms]	*N*_1_ latency [ms]	*N*–*P* amplitude [µV]
Single pulse, 200 µs/phase							
S1	SAN	7.00	21	15.36	10.20	16.60	12.25
S1	LAN	8.00	21	17.16	12.00	16.40	11.60
S2	SAN	8.00	18	12.87	11.80	16.20	10.07
S2	LAN	9.00	17	4.44	9.40	14.80	5.13
S3	PAN	10.00	37	4.65	9.60	16.80	7.09
	Median	8.00	21	12.87	10.20	16.40	10.07
	25th percentile	8.00	18	4.65	9.60	16.20	7.09
	75th percentile	9.00	21	15.36	11.80	16.60	11.60
Single pulse, 50 µs/phase							
S1	SAN	10.00	22	12.49	10.60	15.80	8.86
S1	LAN	9.00	20	15.75	10.80	16.00	11.07
	Median	9.50	21	14.12	10.70	15.90	9.97
	25th percentile	9.25	20.5	12.49	10.65	15.85	9.41
	75th percentile	9.75	21.5	14.94	10.75	15.95	10.52
A train of four pulses, 50 µs/phase, 1600 pps							
S1	SAN	9.00	22	25.05	11.20	16.26	16.26
S1	LAN	9.00	21	20.27	11.60	13.10	13.10
	Median	9.00	21.5	22.66	11.40	14.68	14.68
	25th percentile	9.00	21.25	21.47	11.30	13.89	13.89
	75th percentile	9.00	21.75	23.86	11.50	15.47	15.47

Figure 3 compares the growth functions of the responses of the three vestibular pathways recorded simultaneously and elicited with the single-pulse, 200 µs phase width stimulus profile. To allow comparison between responses, amplitude values were normalized to the maximum obtained per electrode and per patient, for each response. The dynamics of the growth functions were variable across pathways, across subjects, and even across electrodes within subjects. For S1-SAN and S1-LAN, the growth functions of eVOR and ecVEMP responses were practically identical, increasing monotonically with increasing current. Vestibular percept intensities showed a step-like growth function. The responses appeared at the same stimulation current level (i.e., activation thresholds) for the three pathways. In the case of S2-SAN, all pathways were activated in a similar fashion. However, growth functions and were slightly different for the three pathways for S2-LAN, and the eVOR and ecVEMPs were activated at lower currents than that required to elicit a vestibular percept. For S3-PAN, the eVOR pathway was activated at lower currents than the other responses. Vestibular percepts showed the highest activation threshold and showed the steepest growth function.

It is interesting to point out that in the experiments presented here, percept intensities remained relatively low (maximum 3), and therefore, UCL levels were always determined by the maximum current amplitudes allowed by the device. In this particular experimental paradigm, patients did not report any non-vestibular sensations (e.g., sound, pain). Vestibular percepts were generally described as a very slight feeling of motion, balancing, or neck stiffness.

**Fig. 4:**
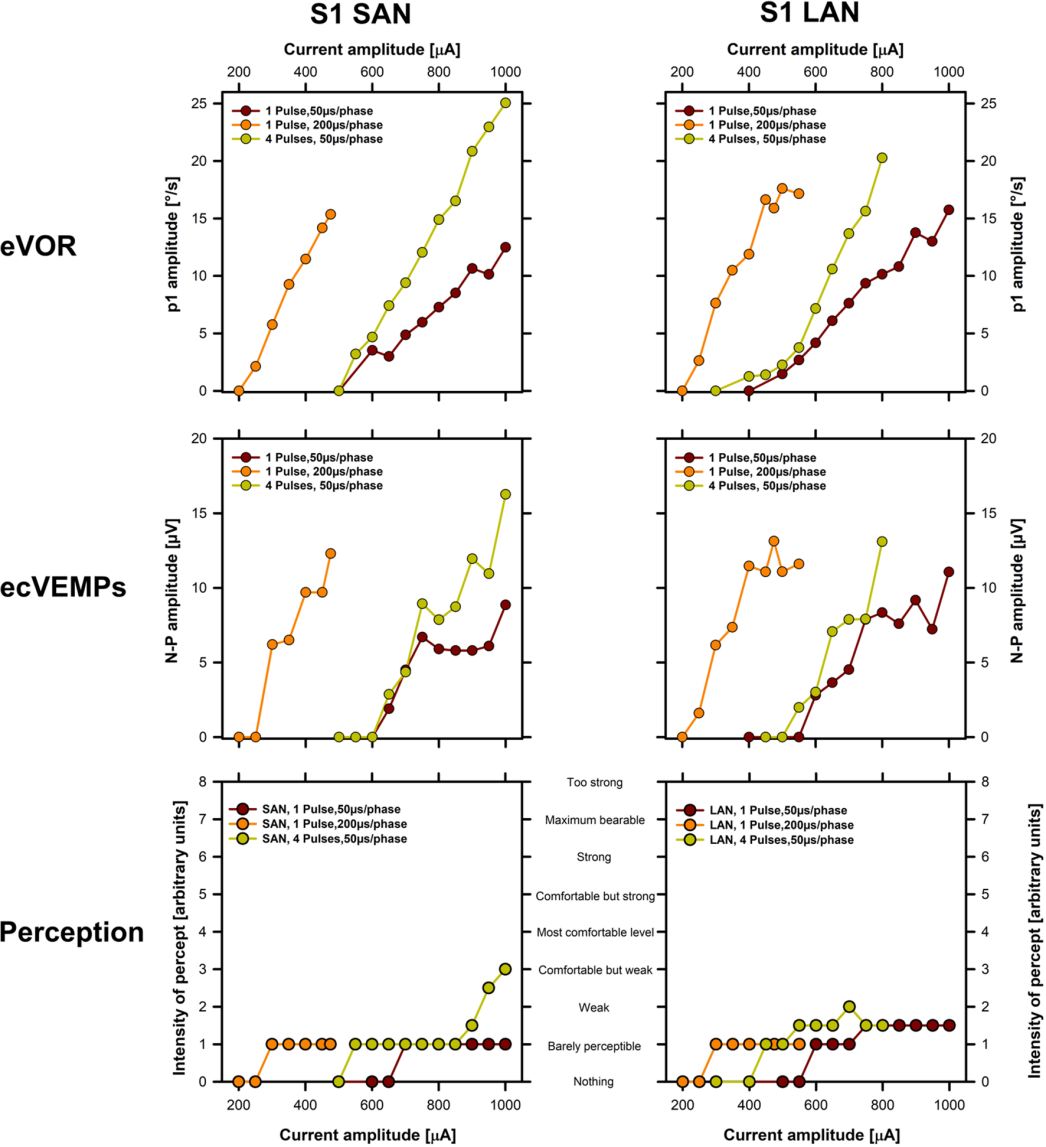
Growth function of the amplitude of the eVOR P1, the ecVEMPs P1 and individual self-reported percept recorded using different stimulation profiles (single pulse, 50 µs per phase—red plot; single pulse, 200 µs per phase—orange plot; train of four pulses, 50 µs per phase, 1600 pps—green plot) versus current amplitude. Only subject S1 was available for this experiment where two electrodes were investigated (electrode SAN—left column and LAN—right column)

**Fig. 5:**
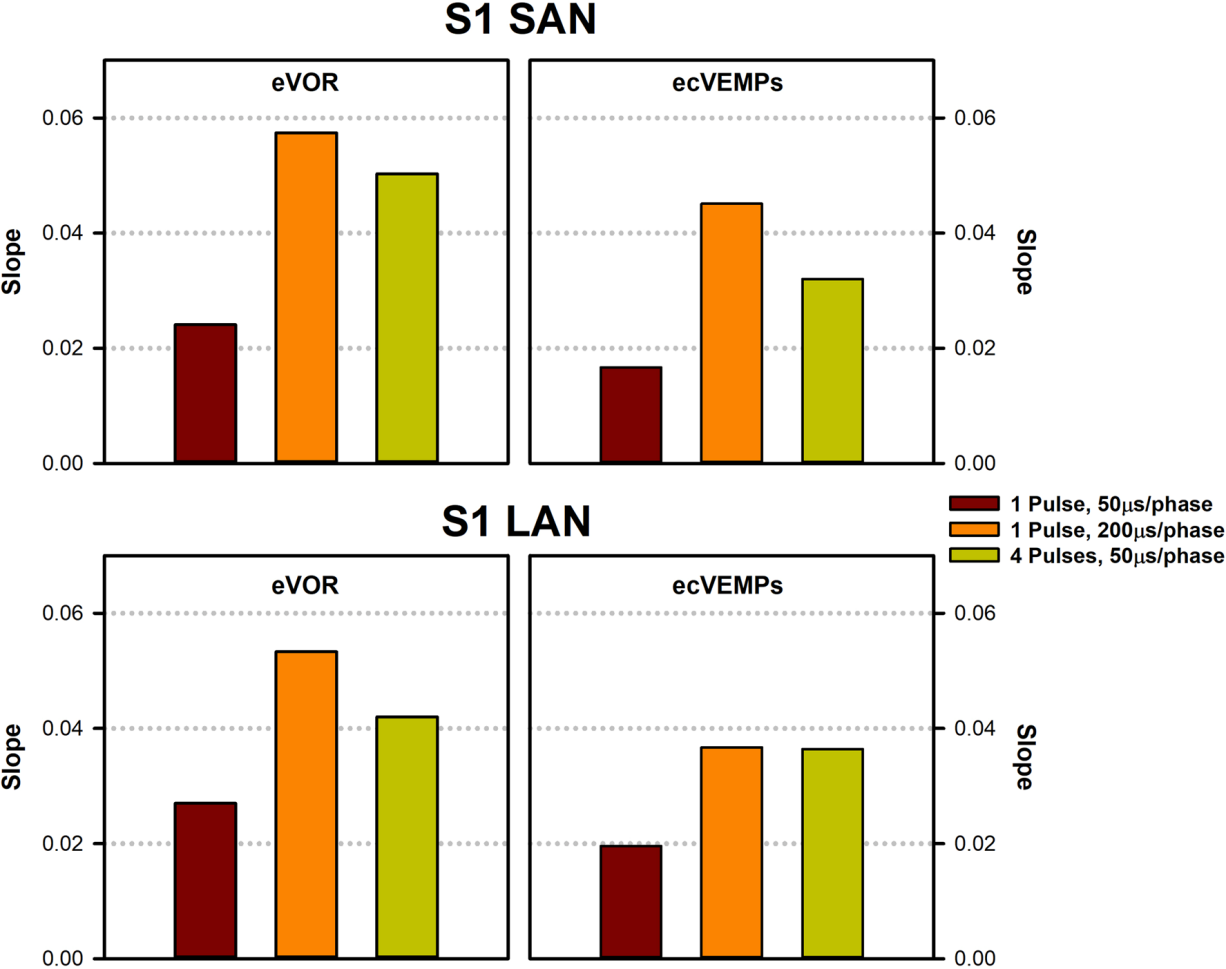
Slope of the growth function of eVOR (left panels) and ecVEMP responses, calculated using linear regression analyses of the normalized data versus stimulation current (see also Fig. 4). Three stimulation paradigms are compared: single pulse, 50 µs per phase—red plot; single pulse, 200 µs per phase—orange plot; train of four pulses, 50 µs per phase, 1600 pps—green plot) versus current amplitude. Only subject S1 was available for this experiment where two electrodes were investigated (electrode SAN—upper line and LAN—lower line). Note that the slope of the growth function for the intensity of percepts was not calculated since it showed a step-like behavior (not a monotonical increase with respect to stimulation current)

The next step of our investigation was to explore the influence of the stimulation profile on all vestibular pathways, measured simultaneously. Only S1 was available for this additional experiment, where stimulation through SAN and LAN electrodes was investigated (Fig. 4). Two phase durations (50 µs and 200 µs) and pulse train durations (single pulse and train of four pulses at 1600 pps) were evaluated. The trend of the results was similar for the SAN (left column) and LAN (right column) electrodes for eVOR and ecVEMP responses. Both reflex responses increased monotonically with increasing current, and a statistically significant correlation was found (Pearson’s linear regression analyses; *p* < 0.001; *R*^2^ > 0.81; slopes shown in Fig. 5). Increasing the phase duration from 50 to 200 µs (with one pulse) resulted in lower activation thresholds and similar peak amplitudes with around half the stimulation current (i.e., doubling the slope of the growth function; red versus orange bars in Fig. 5). Increasing the number of pulses from 1 to 4 for the 50 μs pulse width did not decrease activation thresholds but increased the slope of the growth function by nearly a factor of 2 (red versus green bars in Fig. 5). Peak amplitudes were slightly larger with lower currents for the 4-pulse train. Comparing results for the one-pulse profile with 200 μs pulse width trials to the four-pulse profile with a pulse width of 50 μs (equal charge per stimulation trial) showed that activation thresholds remained lower for the largest pulse width and that the slopes of the growth functions were 10–40% steeper for the 200 µs/phase pulse profile (orange versus green bars in Fig. 5). Maximum peak amplitudes were only slightly larger (0–70%) with the four-pulse profile, but required double the stimulation current.

Overall, perceived intensities remained very low across trials and stimulation currents. The growth function had a step-like behavior, in contrast to the linear behavior of the other pathways. Evoking vestibular percepts required the lowest currents for the single-pulse trials with 200 μs pulse width, and the highest stimulation currents for the single-pulse trials with 50 μs pulse width.

## Discussion

The goal of this study was to explore the simultaneous activation of multiple vestibular pathways. Specifically, we compared their main characteristics (latencies, amplitude growth functions) and explored different stimulation profiles. eVOR, ecVEMPs, and perceptual responses could be evoked in all three tested subjects with five different vestibular electrodes. In this study, reflex pathways showed relatively similar amplitude growth functions that increased monotonically with the amount of charge per stimulus delivered in each case. The amplitude growth function of perceptual responses was different, with perceived intensities remaining quite low and showing a step-like behavior. Finally, the parametric variations attempted here influenced activation thresholds and the growth function of each vestibular pathway. Increasing the phase duration (50 µs vs 200 µs) doubled the slope of the growth function, even when equal charge was delivered per stimulation trial. This might be expected from nerve stimulation at pulse durations well below the chronaxie value (see also [37]).

Response latencies are due to the specific physical constraints of the neural circuitry underlying the corresponding pathways (i.e., synaptic neural delays, neural conduction delays, muscle activation times, and pathway length) [39]. Here we present, for the first time in humans, the compared average latencies of the electrically evoked vestibular reflex pathways. Our results are in accordance with the latencies reported both in human and animal studies for “natural” and electrical stimulation [39–42]. Note that in this study we compared the latency of the initiation of the eVOR (_LAT_eVOR) with the latency of *P*_1_ of the ecVEMPs, because both correspond to the actual initiation of the eye/neck movement. However, in the future, it might be interesting to explore the latencies of the onset of muscular contraction of cervical and ocular myogenic potentials, which could be more informative on the actual time that the signal takes to reach the muscle, providing actual information about the neural pathway without considering the characteristics of the muscle itself.

Comparing the growth functions of the vestibular reflexes shows that, in some cases, the dynamics of the different pathways were not always identical across subjects and that activation thresholds might differ between pathways (Fig. 3). This inter-subject variability may be due to the relative spread of current from ampulla to otolith organs being different in each case. Moreover, the patient profiles were different, which could also explain the differences. For example, while patients S1 and S2 suffered from severe acute bilateral vestibular loss caused by a trauma S3 had a long duration, congenital bilateral vestibular loss. Furthermore, in S3, the active electrode was the PAN electrode (stimulated at an extralabyrinthine location), while in S1 and S2, the stimuli were delivered through the SAN and LAN electrodes (in an intralabyrinthine configuration). However, the small sample size in this study makes it difficult to draw any definite conclusions with respect to the relationship of the location of the active electrodes and the responses.

eVOR and ecVEMP responses increased monotonically with increasing current while perceptual responses showed a step-like growth function (Fig. 4). On the one hand, this observation could be explained because we stimulated with a non-physiological electrical stimulus in isolation (i.e., in the absence of any convergent extra-vestibular stimuli which naturally contribute to perception). This can potentially limit the grow function of intensity perception. On the other hand, this observation could be also explained by the existence of different kinds of vestibular afferent fibers in each vestibular pathway. Morphological and physiological properties of the vestibular afferents (e.g., axon diameter, discharge regularity) allow them to be classified as either regular or irregular [43, 44]. For instance, irregular afferents tend to have thicker axons [45] and thresholds 5–10 times lower than regular afferents [46]. Despite overlap of regular and irregular afferents in each of the subdivisions of the vestibular nuclei [47], electrophysiological studies suggest that the different pathways might be primarily mediated by one type of afferent, with a higher proportion of irregular afferents in the VTC pathway [46]. While these results are still under discussion, these differences in afferent populations combined with the complexity of neuronal connections of the VTC pathway could at least partly explain the observed differences. Moreover, due to the very brief stimulation trials (i.e., the use of 1–4 pulses), the self-perceived stimulation intensities from all patients remained very low, which limited proper evaluation of the growth function. In previous studies [37], where different stimulation profiles and longer stimuli were used, we observed much higher percept intensities. We will attempt to investigate the VTC pathway in more detail in subsequent studies using alternative objective methods (e.g., imaging, electrophysiological) and adapted stimulation profiles.

Finally, to explore the effects of different stimulation paradigms on vestibular responses, three different stimulation profiles were tested on one patient (S1). This investigation revealed that increasing the electrical charge in the stimulus (200 µs vs 50 µs per phase) enhanced all responses (Fig. 4). In addition, while the growth functions of eVOR and ecVEMP responses to two different paradigms containing the same electrical charge (single pulse at 200 µs/phase vs train of four-pulse at 50 µs/phase, 1600 pps) were similar, the slopes were steeper for the single pulse at 200 µs/phase. In other words, a single 200 µs pulse required only half of the current required to evoke an equivalent response with four 50 µs pulses presented at 1600 pps. Therefore, the 200 µs pulse width seems to be a good balance between efficient activation of the vestibular pathways and lower energy consumption. This difference can be related to the physiological features of the nerve. The *absolute* refractory period of a nerve varies between 0.5 and 1 ms and corresponds to the recovery time of the membrane (during which it is impossible to generate additional action potentials). This period is followed by a *relative* refractory period of approximately 10 ms during which the threshold for eliciting spikes is increased. Stimulating with four pulses at 1600 pps means one pulse is applied every 0.6 ms. Consequently, after the first short stimulation pulse, the next might be taking place during the relative refractory period of the nerve which could at least partially explain the observed differences [48].

### Limitations and future work

The main limitation of this study is the small number of patients included, especially for the investigation of different stimulation profiles for which only S1 was available. Indeed, at the present time, only a few patients having received our prototype vestibular implant device are available for testing. This is a common limitation for all current vestibular implant studies (see, e.g., [17, 27, 32]). Furthermore, these experiments are long and time consuming (approximately four hours per patient). We hope that as more patients and more groups get involved in this research, these results will be reproduced in larger cohorts to further validate the observed trends and effects. Note, however, that all subjects showed similar trends. Therefore, despite large intersubject variability, important systematic features could be described.

Overall, the trend between the eVOR and ecVEMPs across different paradigms was similar (Fig. 5). However, the slopes of the eVOR responses seem to be steeper than that of ecVEMPS. While this difference might be related to the strength of activation of each pathway, other parameters inherent to the experiment (e.g.; SCM muscle tension during ecVEMPs recordings) might also have influenced this result.

Another limitation of this study was the evaluation of the VTC pathway through subjective reports of patients and upon very short stimulation trials. Research is currently underway to extend the exploration of these pathways with the use of objective electrophysiological measures.

In summary, this study opens the door to the possibility of selectively activating one pathway or the other, at least in some cases. However, this issue clearly deserves further and more detailed investigation to determine the actual possibilities for selective stimulation, the best strategies, and the functional significance of the contribution of each pathway to the overall rehabilitation process.
